# Convergence of Synapses, Endosomes, and Prions in the Biology of Neurodegenerative Diseases

**DOI:** 10.1155/2013/141083

**Published:** 2013-11-07

**Authors:** Gunnar K. Gouras

**Affiliations:** Department of Experimental Medical Science, Experimental Dementia Research Unit, Wallenberg Neuroscience Center, Lund University, 221 84 Lund, Sweden

## Abstract

Age-related misfolding and aggregation of disease-linked proteins in selective brain regions is a characteristic of neurodegenerative diseases. Although neuropathological aggregates that characterize these various diseases are found at sites other than synapses, increasing evidence supports the idea that synapses are where the pathogenesis begins. Understanding these diseases is hampered by our lack of knowledge of what the normal functions of these proteins are and how they are affected by aging. Evidence has supported the idea that neurodegenerative disease-linked proteins have a common propensity for prion protein-like cell-to-cell propagation. However, it is not thought that the prion-like quality of these proteins/peptides that allows their cell-to-cell transmission implies a role for human-to-human spread in common age-related neurodegenerative diseases. It will be important to better understand the molecular and cellular mechanisms governing the role of these aggregating proteins in neural function, especially at synapses, how their propagation occurs and how pathogenesis is promoted by aging.

## 1. Synapses

The brain is particularly vulnerable to degenerative diseases of ageing. Aberrant aggregation of proteins/peptides is the common theme among these diseases. Alzheimer's disease (AD) and Parkinson's disease (PD) are the most common age-related neurodegenerative diseases, while other less common, albeit devastating, neurodegenerative diseases include Huntington's disease (HD), amyotrophic lateral sclerosis (ALS), prion diseases, and frontotemporal dementia (FTD). Although the specific protein aggregates and selective cellular vulnerabilities differ, shared disease mechanisms are increasingly apparent among neurodegenerative diseases and next to aberrant protein aggregation also include anatomically selective cell-to-cell propagation. Major themes of research on these diseases have included therapeutic neurotransmitter replacement, most successful with dopamine for PD, elucidating the biology of aberrant protein misfolding, and trying to understand how ageing promotes the development of these diseases. More recently, synapses have moved more to the center of research on these diseases [1, 2]. Neurites (axons and dendrites) and synapses are a unique feature of neurons and play fundamental roles in brain function. Furthermore, the aggregation-prone proteins linked pathologically and genetically to neurodegenerative diseases are normally present particularly at synapses. For example, the PD-linked protein *α*-synuclein is known to normally reside primarily in presynaptic compartments [3, 4], although, as the name indicates, a nuclear role also characterizes this protein that aggregates in the distinctive cytoplasmic Lewy bodies and Lewy neurites that characterize PD and the related Lewy body dementia (LBD). 

**Figure 1:**
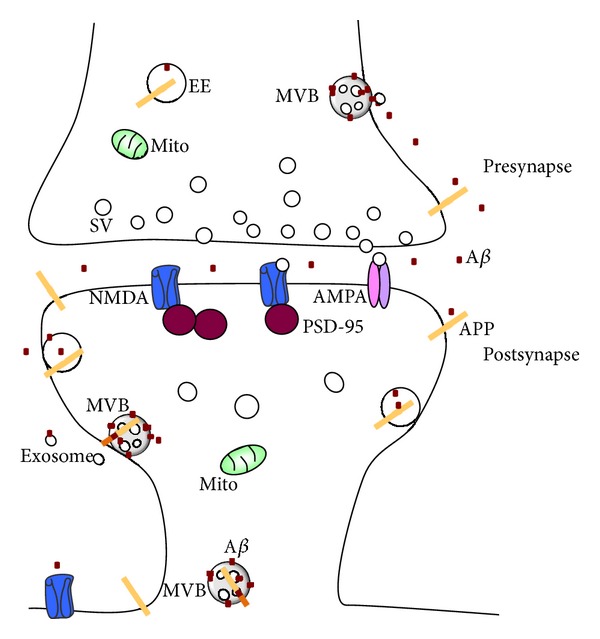
Schema of synaptic biology relating to Alzheimer's disease. APP is present in endosomes, including early and late/MVB endosomes, as well as at the cell surface. A*β* is associated with MVBs and other endosomes, as well as being secreted from the cell surface, also via exosomes. The relative proportions of A*β* peptides and APP processing in the pre- versus postsynapse remain uncertain. The cellular mechanism(s) of A*β* transmission from or to the pre- and postsynapse is also not yet clear. MVB: multivesicular body; EE: early endosome; SV: synaptic vesicle.

An important role at synapses for the AD-linked *β*-amyloid (A*β*), and the amyloid precursor protein (APP) from which it is derived, is also increasingly becoming apparent (Figure 1). APP is transported down axons and dendrites to synapses [2, 5], where the proteases that generate A*β* are also localized [6]. The precise processing and trafficking of APP and A*β* in pre- versus postsynaptic compartments and how these relate to the mechanism of synaptic damage in AD remain to be elucidated. Evidence supports that A*β* accumulation in synapses alters synaptic function by altering important synaptic proteins and receptors [2].

A major hurdle for research on neurodegenerative diseases has been that the normal physiological roles and functions of the aggregation-prone proteins have been difficult to ascertain. A potential reason for this could very well be that synapses are so complex and are only gradually being elucidated. Although mouse knockout studies do not support that loss of function of the disease-linked proteins is the salient issue in these various diseases [7, 8], it is nevertheless possible that their propensity to aggregate is a feature that makes these proteins normally important at synapses. It is further possible that a better understanding of the normal function of neurodegenerative-linked proteins at synapses will be important in order to uncover better therapeutic targets and devise more effective therapies for these diseases.

Synaptic activity and plasticity are of central importance in the brain and at synapses, and it has become clear that neurodegenerative disease-linked proteins are modulated by synaptic activation [9, 10]. The major nonneuronal cells of the brain, the astrocytes and microglia, are also increasingly linked to synaptic function and thereby might impact the pathophysiology of these diseases that appear to initiate at synapses. Modulation of synapses has also been shown to directly impact synapse damage in the brain of transgenic mouse models of neurodegenerative diseases [11].

## 2. Endosomes

The endosome-lysosome system and the ubiquitin proteasome system (UPS) play many essential roles in cells and are increasingly implicated in neurodegenerative diseases of ageing [12]. In neurons, these systems, best known for their role in protein degradation, are also important for the normal function of synapses [13]. The diversity of rare genetic neurodegenerative storage diseases of childhood linked to aberrant protein or lipid accumulation in the endosome-lysosome system supports the potential disease relevance of this system also in the common age-related degenerative diseases of the brain [14]. The endosome-lysosome system is involved in many central functions, including cellular internalization, degradation, and release. In Down syndrome (DS), characterized by trisomy of chromosome 21, which invariably leads to age-related AD-like pathology and dementia, abnormal endosome enlargement has long been known to precede the characteristic neuropathological amyloid plaques and tau tangles [15].

The related autophagy system is intimately linked with the endosome-lysosome system and is important for the engulfment and degradation of larger subcellular structures, including whole organelles. Autophagy is a prominent neuropathological feature of neurodegenerative diseases [16], where autophagic vesicles, limited by double or multilamellar membranes, accumulate in neurons and their processes. Autophagic vesicles are particularly abundant in dystrophic neurites in AD brain and transgenic mouse models of AD. Autophagic vesicles are thought to form from the formation of double membranes in the cytoplasm. Autophagic vesicles are thought to subsequently fuse with endosomes or lysosomes followed by degradation of their contents by lysosomal proteases. Although autophagy is considered a mechanism of normal cellular degradation, autophagy is rarely seen on electron micrographs of normal brain (personal observations). The autophagy system is further implicated in neurodegenerative diseases of ageing, since inhibition of the mammalian target of rapamycin (mTOR), which is well known to induce autophagy, was found to prolong the lifespan in lower organisms and mammals [17]. More recent studies show that pharmacological mTOR inhibition by rapamycin is protective in transgenic mouse models of Alzheimer's disease [18, 19]. A challenge for the development of treatments of neurodegenerative diseases based on mTOR inhibition is that mTOR is part of a central signaling system regulating many cellular functions. For example, mTOR is important in synaptic plasticity [20].

Neurites can extend considerable distances and synapses are thus at a greater distance from their cell bodies than are components of other cells. Moreover, lysosomes do not normally reside in neurites and synapses. Therefore, protein degradation at synapses depends on retrograde transport via endosomes back to the cell body. Late endosomes can take on some limited lysosomal functions, but compared to lysosomes they are much less efficient at degradation, particularly of aggregation prone insoluble proteins. Endosomes also play important roles in the regulated delivery, recycling, and degradation of receptors at synapses that are important for synaptic plasticity [21]. Interestingly, the inner vesicles that characterize the late endosomal multivesicular body (MVB, alternatively called multivesicular endosome) are released as exosomes, and increasing evidence suggests that exosomes may be important in the release and propagation of neurodegenerative disease-linked proteins [22]. Several of these disease-linked proteins have been localized to endosomes. For example, AD-linked A*β* peptides were shown to normally localize and then accumulate and aggregate particularly in MVBs of dystrophic neurites and synapses of AD transgenic mouse models and human AD brains, where they associated with localized subcellular pathology, even prior to extracellular amyloid plaques [23–25]. 

The UPS is also increasingly linked with neurodegenerative diseases of ageing. For example, mutations in Parkin represent an example of a UPS-linked protein (component of a ubiquitin ligase) that is mutated in familial forms of PD. Moreover, the UPS and the endosome-lysosome system are linked, since transmembrane proteins at the cell surface, including APP and neurotransmitter receptors at synapses, can be ubiquitinated and routed via the endocytic pathway to the lysosomes for degradation [13, 26]. AD-linked A*β* normally localizes to and with AD pathogenesis preferentially accumulates at the outer limiting membranes of MVBs [23] where the endosomal sorting complexes required for transport (ESCRT) reside, which is involved in targeting ubiquitinated transmembrane proteins for degradation. Experimental evidence supports that accumulating A*β*42 associated with MVBs impairs MVB sorting by disrupting the UPS [26]. Altered regulation of synaptic protein trafficking from early aberrant protein/peptide accumulation near synapses might lead to the earliest synapse dysfunction in neurodegenerative diseases [2]. Interestingly, mutations of CHMP2b, a component of ESCRTIII, have been linked with familial forms of FTD [27]. Although FTD, the 2nd most common cause of dementia before the age of 65, is not genetically linked with A*β*/APP, it has been linked to familial mutations in tau, the main constituent of the other characteristic neuropathology of AD, the neurofibrillary tangles. Moreover, cells release tau, which interestingly is also stimulated by synaptic activity [28]. Release of tau may potentially occur via MVBs and exosomes. Cell-to-cell propagation of tau has recently become a hot topic in the field of neurodegenerative diseases [29]. 

The endocytic pathway is also central to cholesterol and lipid uptake and trafficking in neurons, which are implicated in AD [30, 31] and are important in injury and synapse remodeling in neurons. The lipoprotein carrier apolipoprotein E4 (ApoE4) polymorphism is the major genetic risk factor for typical late onset AD. Cholesterol traffics via the endocytic pathway into cells, and together with the endoplasmic reticulum (ER) and mitochondrial-ER membranes, this interconnected pathway that regulates cellular cholesterol metabolism has been related to A*β* [32]. Recent research supports that apoE, generated mainly by astrocytes, is important for regulating neuronal A*β* [33, 34]. Interestingly, the apoE receptor, low-density lipoprotein receptor (LDLR) related protein (LRP), is routed from the plasma membrane to the limiting membrane and then internal vesicles of MVBs and interacts with APP via Fe65 [35]. As noted previously, A*β* normally localizes to MVBs and with AD pathogenesis accumulates at MVBs, and upon release from the cell, MVB inner vesicles are then called exosomes. Thus, it is conceivable that apoE biology intersects with A*β*/APP in endosomes and might even modulate propagation of secreted misfolded proteins via exosomes and/or protein degradation via the MVB-lysosome pathway. In addition, endosomes have a lower pH, which is known to promote aggregation and amyloid formation of misfolding proteins, including A*β* and PrP [36–38]. Finally, evidence consistent with leakage of endosome-lysosome contents by A*β* has been reported [39, 40]. Consistent with such leakage, immunoelectron microscopy showed marked accumulation of A*β*42 directly associated with outer membrane disruption of MVBs in AD transgenic mice [23]. Thus, multiple lines of evidence point to an important role of the endosomal-lysosomal system and in particular of endosomes at neurites and synapses in common neurodegenerative diseases of ageing.

## 3. Prions

Prions are the unusual proteins that are best known for their ability to propagate disease between members of a species and between different species [41]. Prion diseases have therefore been classified among infectious diseases. Formerly classified as atypical “slow virus” diseases, this nomenclature was abandoned, since they propagate as proteins and in contrast to viruses lack nucleic acids. However, clinically and pathologically, prion diseases are most similar to common neurodegenerative diseases, such as AD. Prions can form fibrillar aggregates with amyloid-like characteristics. The normal role of the prion protein is also poorly understood [8], although it is expressed at particularly high levels in the brain and is thought to primarily localize to synapses. Analogous to AD and PD, the majority of prion diseases are sporadic, without a clear infectious or genetic cause. Less common familial forms of prion disease with mutations in the prion protein exist. Although the term prion is widely associated with fear, based on its infectivity from highly publicized outbreaks of prion disease, prion proteins are normal proteins that can even provide protective functions [42, 43]. Thus, prions, like amyloids [44], should not be only viewed in a negative light, and PrP likely plays important physiological roles, which potentially may be particularly relevant at synapses. The mere evolutionary presence of PrP, which is conserved in mammals, as well as some deficits noted in PrP deficient mice [45], supports a physiological role for PrP. A more recent development in research on neurodegenerative diseases is the surprising realization that other disease-linked aggregation prone proteins, such as A*β* [46, 47], *α*-synuclein [48, 49], and tau [29], can also propagate in experimental systems. 

Despite the many years of research on prion propagation, the cellular mechanism(s) of propagation still remain(s) unclear. The recent surge of research on this topic particularly in AD, PD, ALS, and HD will undoubtedly lead to important new insights. Some overlooked earlier papers have provided clues to cellular mechanisms of propagation in these diseases. For example, a literature from the 1990s showed that exogenously added extracellular AD-linked A*β*1–42 peptides induced a marked upregulation of newly generated A*β*42 within the treated cells [50]. Moreover, it was shown that extracellular A*β*1–42 failed to alter synapses in the absence of APP or in neurons where de novo generation of A*β* was inhibited [10]. Similarly, depletion of endogenous PrP protects against scrapie-induced PrP pathogenesis [51, 52]. While neurons generate A*β* from APP within neurons, secreted A*β* can also be internalized by neurons [53]. More recent evidence points to synapses as selective sites of neuron-to-neuron spread of A*β* [54]. One could speculate that release of exosomes might be particularly prominent near synaptic terminals, although it was estimated that only about 1% of released A*β* was associated with exosomes [55]. Overall, the cell biology of such synapse-associated endocytic and exocytic pathways in neurons is less well understood than of other cells.

A challenge in considering prion-like cell-to-cell propagation is to explain where and how the initial pathological conformation of disease-linked peptide forms and what determines the anatomical selectivity of spread by various disease-linked proteins. Recent evidence supports the surprising scenario that in the setting of aberrant intracellular protein aggregation, the secretion of AD-linked A*β* is actually impaired [56]. In general, the release of more toxic soluble oligomers appears to be at much lower levels than those of monomeric proteins; for example, the concentration of A*β* oligomers is about 1% of the monomeric forms in cerebrospinal fluid. In addition, aggregation of misfolded proteins begins in a selected population of neurons. Thus, it follows that if abnormal aggregation can initially arise spontaneously in one anatomical region, it might be possible that other vulnerable cells might also have de novo appearance of abnormal aggregated conformations rather than a prior requirement for propagation from other cells. It is also possible that despite the reductions in the normal secretion of monomers, cell-to-cell transmission of more aggregated oligomers may be the driving force in disease propagation, which even at low levels might still act as the nidus to drive further aggregation in the recipient cells.

There is no convincing evidence that proteins linked to the common age-related neurodegenerative diseases can spread from person to person as was recently highlighted in a study of patients who had received growth hormone [57]. Thus, neuroscientists need to contribute to reducing the excessively alarmist view that is linked with the term prion in the public. Actual prion diseases are remarkably rare in humans and even the highly publicized outbreaks were relatively small in scale. For example, the number of human cases of variant Creutzfeldt-Jakob disease cases per year associated with the outbreak of bovine spongiform encephalopathy in Great Britain peaked in the year 2000 with 28 deaths (http://www.promedmail.org/direct.php?id=20120809.1236446). Yet, this overall low incidence of human cases should not take away from the importance of proper safety precautions associated with avoiding contamination with prions or in efforts to prevent prion outbreaks in animals and man.

## 4. Conclusion

Neurodegenerative diseases of ageing are a growing disease epidemic that is placing an increasing financial and emotional toll on societies. Our slow progress in developing treatments that will eventually slow down or even halt the progression of these debilitating diseases likely hinges on a better understanding of the complexity of the ageing brain, cell biology, and synapses. Rare gene mutations or more common polymorphisms have provided new clues in our understanding of the biochemical pathways that determine these important diseases. Synapses are turning out to be potentially critically vulnerable sites prone to diseases of protein misfolding. The major degradative organelles, the lysosomes, localize to the cell body of neurons and are thus removed from distal neurites and synapses in which various endosomal organelles provide diverse functions, including secretion and degradation. Cellular degradation systems, such as the endosome-lysosome system and the UPS, may be particularly vulnerable to the development of age-related dysfunction of the nervous system and thereby might predispose to aberrant protein aggregation with ageing. Major contributors to the ageing process, including mitochondrial dysfunction, cardiovascular disease, and inflammation, likely impact the declining function of these important cellular degradation pathways. It will be critical to better define the cellular and biochemical pathways implicated in neurodegenerative diseases as well as to elucidate the normal biology of synapses. It is also possible that the aggregation-prone properties of misfolding proteins linked to neurodegenerative diseases hinge on their normal role at synapses and their propensity to aggregate. Furthermore, it will be important to better define the more precise cellular mechanisms leading to cell-to-cell propagation of neurodegenerative disease-linked proteins.
